# Magnetic map navigation in a migratory songbird requires trigeminal input

**DOI:** 10.1038/s41598-018-30477-8

**Published:** 2018-08-10

**Authors:** Alexander Pakhomov, Anna Anashina, Dominik Heyers, Dmitry Kobylkov, Henrik Mouritsen, Nikita Chernetsov

**Affiliations:** 1 Biological Station Rybachy, Zoological Institute of Russian Academy of Sciences, 238535 Rybachy, Kaliningrad Region Russia; 2 Sechenov Institute of Evolutionary Physiology and Biochemistry, Russian Academy of Sciences, 194223 St. Petersburg, Russia; 3 Arbeitsgruppe “Neurosensorik/Animal Navigation”, Institut für Biologie und Umweltwissenschaften, Universität Oldenburg, D-26111 Oldenburg, Germany; 4 Research Centre for Neurosensory Sciences, University of Oldenburg, D-26111 Oldenburg, Germany; 5 Department Vertebrate Zoology, St. Petersburg State University, 199034 St. Petersburg, Russia

## Abstract

Recently, virtual magnetic displacement experiments have shown that magnetic cues are indeed important for determining position in migratory birds; but which sensory system(s) do they use to detect the magnetic map cues? Here, we show that Eurasian reed warblers need trigeminal input to detect that they have been virtually magnetically displaced. Birds with bilaterally ablated ophthalmic branches of the trigeminal nerves were not able to re-orient towards their conspecific breeding grounds after a virtual magnetic displacement, exactly like they were not able to compensate for a real physical displacement. In contrast, sham-operated reed warblers re-oriented after the virtual displacement, like intact controls did in the past. Our results show that trigeminally mediated sensory information is necessary for the correct function of the reed warblers’ magnetic positioning system.

## Introduction

To perform true navigation, migrating animals need both a compass and a map^1–5^. There has been quite some controversy whether migrating birds use a map based on gradients of geomagnetic field parameters for long-distance navigation^3,6–8^. We have recently been able to show that Eurasian reed warblers (*Acrocephalus scirpaceus*) use a magnetic map during their annual long-distance movements^9,10^, like sea turtles^11,12^, newts^13^, salmonid fish^14,15^ and eels^16^ do. The aim of the present study was to discover which magnetosensory system night-migratory birds use to navigate with their magnetic map. Previously, we showed that Eurasian reed warblers could only compensate for a physical west-east displacement if the ophthalmic branches of their trigeminal nerves (V1s) were intact; individuals that had their V1s bilaterally ablated continued to orient in the seasonally appropriate migratory direction but did not compensate for a displacement across longitude^17^. As V1s are known to transmit, among other, magnetic information to the brain^18–20^, we hypothesized that it might be the magnetic information that is used by Eurasian reed warblers for navigation. However, since we did not specifically manipulate magnetic cues in the real displacement experiments, we could not demonstrate that magnetic information was used/blocked. To critically test this hypothesis, it was therefore necessary to perform a virtual magnetic displacement study^9^, in which the magnetic information was the only parameter changed while all other potential cues remained unaltered. The results of this crucial study are reported here.

**Figure 1:**
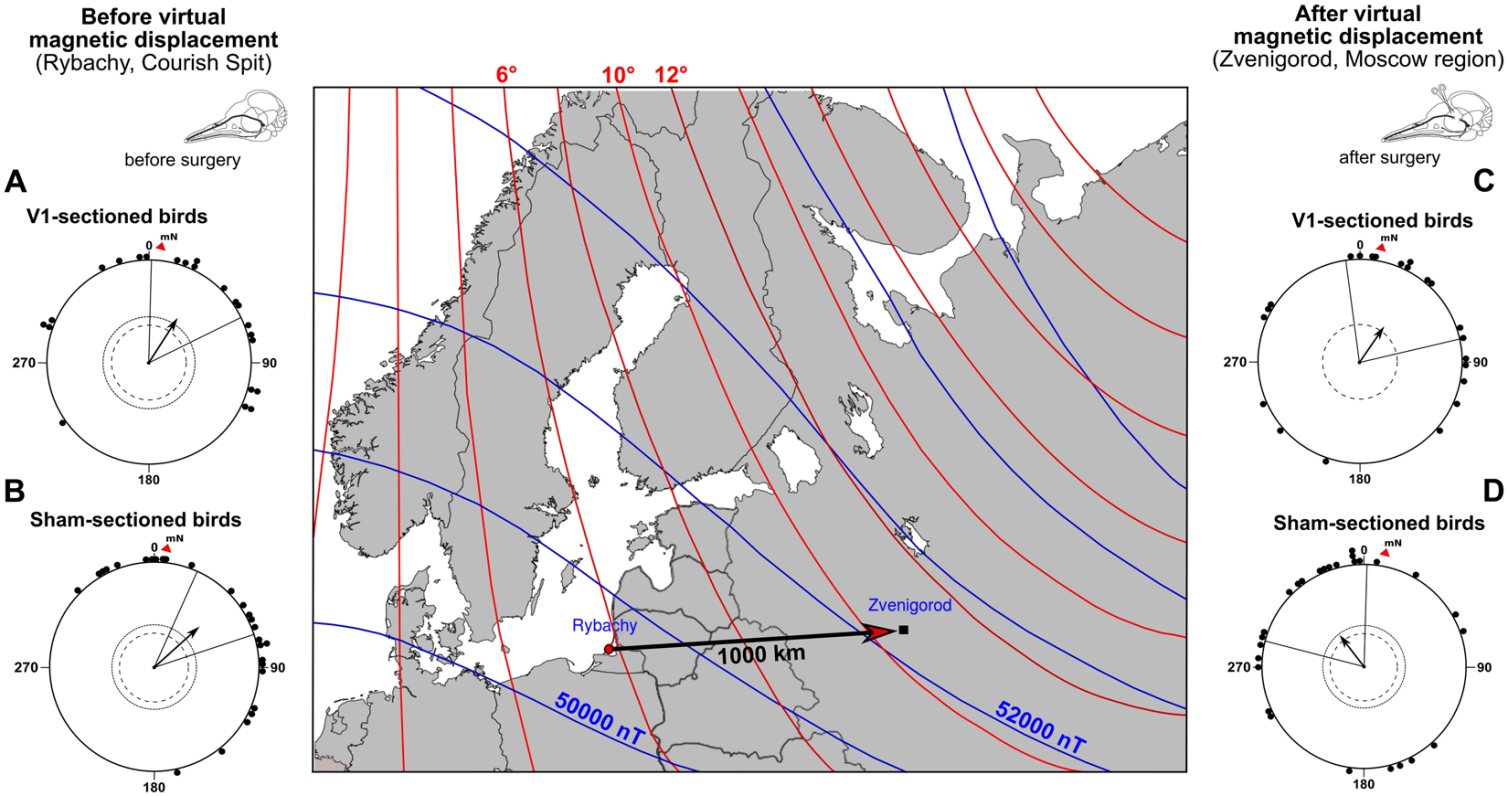
Centre: map of natural magnetic field parameters of the capture site (Rybachy, Kaliningrad region) and the site of virtual displacement (Zvenigorod, Moscow region). The solid arrow on the map shows the virtual displacement direction and distance. The red and blue lines on the map are the magnetic declination and magnetic intensity isolines, respectively. The circular diagrams show the orientation of the Eurasian reed warblers tested at the capture site before surgical treatment and virtual displacement ((**A**) real V1-sectioned group, (**B**) sham-sectioned group) and the same birds’ orientation after a purely magnetic 1,000 km virtual eastward displacement ((**C**) V1-sectioned group, (**D**) sham-operated group). Each dot at the circle periphery indicates the mean orientation of one individual bird; arrows show mean group vectors; the dashed circles indicate the radius of the group mean vector needed for 5% and 1% levels of significance according to the Rayleigh test of uniformity; solid lines flanking mean group vectors give 95% confidence intervals for the group mean directions. Geographic North corresponds to 0°, magnetic North (mN) is shown by a red triangle outside the circle (+5.5° declination in Rybachy and +10.1° declination in Zvenigorod). The map was produced in R 3.2.5 (R Foundation for Statistical Computing, http://www.R-project.org/) using packages “map”, “mapproj” and “mapdata”.

We captured 49 Eurasian reed warblers during spring migration in Rybachy (55°09′ N, 20°52′ E, Fig. 1) and housed them in wood and cloth-net outdoor cages located inside a three-dimensional double-wrapped Merritt 4-coil system which allowed us to accurately and homogenously change any magnetic field parameter^21,22^. The birds’ spontaneous migratory orientation was recorded in Emlen funnels^23^ placed on top of the housing cages during testing. The birds never left the highly homogeneous magnetic field area in the centre of the coil system. Both the housing cages and the Emlen funnels provided the birds with free access to all non-magnetic cues including unaltered photoperiodic, celestial, olfactory, and landmark cues^10^.

## Results

When we tested Eurasian reed warblers in the natural magnetic field of Rybachy (total magnetic field intensity 50,118 nT, inclination 70.1°, declination +5.5°), they oriented in their seasonally appropriate NE direction (*α* = 42° [all directions in this paper are indicated relative to geographic north], *r* = 0.54, *n* = 49, *P* < 0.001, 95% confidence interval of the group mean direction [CI_mean] = 22°–61°). This result fits exactly to the mean orientation of Eurasian reed warblers tested over the previous decade in Rybachy (α = 42°, r = 0.46, *n* = 52; *P* < 0.001, CI_mean = 19°–64°)^15^.

After the orientation tests at the capture site, and prior to the virtual magnetic displacement, we split the birds into two groups with very similar circular distributions. In one group, the ophthalmic branches of the trigeminal nerves were cut bilaterally (V1 sectioned birds, Fig. 1A, orientation of these individuals in Rybachy: *α* = 33°, *r* = 0.51, *n* = 22, *P* = 0.003, CI_mean = 1°–64°). In another group, the same surgical treatment was undertaken but the ophthalmic branches of the trigeminal nerves were left intact (sham-sectioned birds, Fig. 1B, orientation of these individuals in Rybachy: *α* = 48°, *r* = 0.58, *n* = 27, *P* < 0.001, CI_mean = 24°–72°). These are the same data as reported in the previous paragraph, but split into two groups according to the experimental treatment the birds had been assigned to: real ablated or sham-operated. The experimenters did not know which birds had undergone which surgery until after all orientation tests had been completed.

Three to seven days after the surgery, we virtually magnetically displaced the birds by continuously keeping and testing them inside the 99% homogeneity volume in the centre of a three-axial Merritt-4-coil system^24^ set to generate a magnetic field identical to that found in Zvenigorod near Moscow, 1000 km east of their real location (55°42′ N, 36°45′ E; total intensity 52,175 nT, inclination: 71.2°, declination +10.1°). Previous studies have shown that real physical displacements to that location results in re-orientation consistent with correcting for the displacement^17,25^. For detailed experimental procedures, see Methods.

Sham-operated Eurasian reed warblers compensated for the virtual magnetic displacement to Zvenigorod by re-orienting their compass orientation towards NW (Fig. 1D; α = 323°, r = 0.39, *n = *27, *P* < 0.017; CI_mean = 284°–1°). Their orientation was significantly different from the orientation in the local Rybachy magnetic field before the same individual birds underwent the sham surgery (Mardia-Watson-Wheeler [MWW] test: W = 10.9, *P* = 0.004). In contrast, Eurasian reed warblers whose V1s had been bilaterally sectioned did not re-orient towards their breeding areas after virtual magnetic displacement to Zvenigorod (Fig. 1C; α = 34°, r = 0.39, *n = *22, *P* < 0.036; CI_mean = 352°–76°). In fact, the orientation of these birds after virtual displacement was statistically indistinguishable from their orientation in the local Rybachy magnetic field before they underwent surgery (MWW test: W = 0.019; *P* = 0.99; 95% CI overlap completely). Furthermore, after the virtual magnetic displacement, the sham-operated Eurasian reed warblers were significantly more westerly oriented than the real V1-ablated birds were (MWW test: W = 7.66, *P* = 0.022).

## Discussion

We have previously shown that virtual changes in magnetic parameters alone^9^ are sufficient to elicit a re-orientation response in Eurasian reed warblers towards their breeding destinations, which is indistinguishable from the re-orientation observed after a real physical 1000 km eastward displacement^25^. In the present experiments, the birds were not physically but magnetically moved in a sort of magnetic virtual reality. All other potential navigational parameters including photoperiodic, celestial, olfactory, and landmark cues remained unchanged and thus indicated that the birds had not been displaced. We can therefore be sure that the re-orientation response in the sham sectioned birds was caused by magnetic field parameters. Since the birds with cut V1s were unable to re-orient, we unequivocally show that the ophthalmic branch of the trigeminal nerve carries at least some part of the information which is essential for the birds’ magnetic map. Any alternative explanation, e.g. that V1 ablation blocked the expression of a *Zugknick* (a programmed change in migratory direction independent of current magnetic or any other conditions), is highly unlikely. First, no *Zugknick* from NE to NW in spring occurs in Eurasian reed warblers^26,27^. Second, testing of sham and real ablated birds was done at the same time of the year (see Methods), so even if a *Zugknick* would have existed, the sham and real lesioned group should be affected equally in their directionality, but they in fact showed different orientation directions after the virtual displacement (see Fig. 1). Third, we performed our experiments in spring, when long-distance migrants are generally believed to navigate towards their breeding destinations rather than to follow an inherited spatiotemporal programme^3,4,28^. Fourth, it is not clear how V1 ablation should interfere with any inherited programme used in spring, except by blocking magnetic information input, which normally triggers re-orientation^9^. This reorientation would require use of a magnetic map as we are suggesting. The possibility that V1-ablations caused neuropathic pain that prevented the reed warblers from being able to use their navigational map is similarly unlikely. Orientation in Emlen funnels is based on spontaneous nocturnal activity that healthy, non-stressed, caged migrants express in captivity. If the birds are in pain or otherwise strongly affected, they normally do not express any nocturnal migratory restlessness (*Zugunruhe*), and certainly not predictable directionally oriented migratory restlessness in Emlen funnels. By far the most parsimonious explanation for our data is that V1 carries magnetic map information which is needed for the birds’ ability to determine their location relative to their goal.

Considering that experienced reed warblers can use magnetic declination (the difference in direction towards geographical North and geomagnetic North), which requires the detection of both magnetic and celestial compass information, to solve the longitude problem^2,10^, our data strongly indicate that both trigeminally and visually perceived magnetic information is required for the magnetic map to work.

It has been shown that the trigeminal brainstem complex, which is innervated by V1, is activated by magnetic fields in night-migratory European robins (*Erithacus rubecula*)^18^ and Northern wheatears (*Oenanthe oenanthe*)^20^. Taken together, the present data strongly support the hypothesis that V1 innervates magnetic sensors and that these sensors provide a crucial part of the information needed for the magnetic map of long-distance night-migratory songbirds. The identification of the magnetic sensors innervated by V1 remains one of the most important challenges in the study of orientation and navigation mechanisms in migratory birds^29–32^, which are crucial to understand when designing conservation measures for avian migrants^33^.

## Methods

### Experimental birds and sites

We captured 49 Eurasian reed warblers during their spring migration during May 2013–2015 and 2017 at Rybachy on the Courish Spit (sex unknown, the sample probably included both second calendar year birds and older individuals). The birds were provided with food (mealworms) and water ad libitum and were kept outdoors in individual cages (40 × 30 × 30 cm; see photo^9^), which provided them with a clear view of celestial orientation cues and the cages had good air circulation so that local Rybachy olfactory information was continuously available both before and after the virtual magnetic displacement^10^. Due to the remote nature of the location (11 km from the nearest human settlement), it is almost certain that the level of anthropogenic electromagnetic noise was far below the level that can disrupt the birds’ magnetic compass^22,34^, as indicated by numerous orientation experiments performed with night-migratory songbirds at this location previously^9,10,17,25,35^.

All animal procedures were approved by the appropriate authorities: permit 2013–08 by Kaliningrad Regional Agency for Protection, Reproduction and Use of Animal World and Forests; and permit 2014–12 by the specialized committee of the Scientific Council of the Zoological Institute, Russian Academy of Sciences in Russia. Permits to do identical experiments were also approved by the equivalent German authorities (LAVES). All experiments were performed in accordance with relevant guidelines and regulations

### Manipulations of the magnetic field and testing procedure

The birds’ orientation responses were tested in modified Emlen funnels^23^ made of aluminium (top diameter 300 mm, bottom diameter 100 mm, slope 45° with the top opening covered by netting). The Emlen funnels provided access to all natural cues including celestial and olfactory cues. All Emlen funnel tests were performed within a double-wrapped, three-dimensional Merritt four-coil system^24^. The coil system was identical to the ones used by the Oldenburg group and described in detail elsewhere^21,24,36^. First, the birds were tested in the natural geomagnetic field of Rybachy. The individuals which showed significantly directed orientation (in any direction) in the Rybachy field were subsequently placed into an experimentally modified magnetic field, whose parameters were adjusted to be identical to the natural geomagnetic field at Zvenigorod Biological Station of Moscow State University, ~40 km W of Moscow (55°42′ N, 36°45′ E). The Zvenigorod magnetic field was produced when electric current ran in the same direction through the two subsets of windings of the double-wrapped, three-dimensional Merritt four-coil system^24,36^. During the preceding tests in the natural field of Rybachy (control tests), the same current was run antiparallel through the two subsets of windings.

The birds were magnetically displaced only once and were housed inside the magnetic coil system 24 h/day in the changed magnetic field throughout the testing period, which lasted for several days. This was achieved by keeping the birds in cages arranged in the lower half of the 99% homogeneity area within the coils and testing the birds in the upper half of this area (see photo^9^). This ensured that the birds never left the 99% homogeneity area of the magnetic coil system during the entire treatment period, and multiple simulated displacements were thus avoided. The “99% homogeneity area” refers to the area within the coils, where the heterogeneity of the additional field component generated by the coils is <1%. In the present case, the field generated by the coils was ca. 2000 nT, which means that the variability generated by the coils within the birds’ living and testing space was <±20 nT in the present experiments, and thus smaller than the typical daily stochastic variations in the geomagnetic field^3^.

All tests were performed at night when the glow from the setting sun was minimal and exactly in the north (in tests later in the migratory season the glow never ceased completely). Each test lasted 40 min and tests were only performed when at least 50% of the starry sky was visible; in most tests, the sky was 95–100% clear. The directionality of the birds’ activity was recorded as scratches left when birds were hopping in the funnels on a print film covered with a dried mixture of whitewash and glue. Two researchers independently determined each bird’s mean direction from its distribution of scratches. If both observers considered the scratches to be randomly distributed or if the two mean directions deviated by more than 30°, the bird was considered as disoriented in a given test. The mean direction of all the individual bird’s directions was recorded as an orientation data point. We included the results of those birds that were tested at least three times and that showed at least two tests being sufficiently active (i.e. left at least 40 and nearly always >100 scratches on print film) and with orientation sufficiently concentrated according to the Rayleigh test at 5% significance level. If a bird was inactive (fewer than 40 scratches) or disoriented (the mean vector not significant) in a particuar test, the results of this test were excluded from the analysis (see Supplementary Tables S1 and S2 for the results of all individual tests). The group mean vectors for each condition were calculated by vector addition of unit vectors in each of the individual birds’ mean directions^37^.

The nonparametric Mardia-Watson-Wheeler test, which tests whether two samples are different from each other with respect to mean directions, variance, or a combination of both measurements^37^, was used to test for differences in orientation between experimental groups. We did not use the more powerful parametric Watson-Williams test because the *r-*values for our group mean vectors in most cases were <0.75. An *r* value > 0.75 is a crucial assumption for this test^37^. Results were regarded as significant if *P* < 0.05. Statistical tests were performed with ORIANA (version 3.21). We also compared 95% and/or 99% confidence intervals of the mean directions between groups.

Eurasian reed warblers that migrate through Rybachy in spring have their breeding destinations in the Baltic countries, Finland and NW Russia, as per ringing recaptures^26,27^. Therefore, the birds virtually displaced to Zvenigorod were most probably southeast of their migratory destinations, and if they compensate for the simulated displacement they should show northwesterly orientation when tested in Emlen funnels, like the physically displaced intact^25^ and sham-operated^17^ conspecifics and virtually displaced intact birds^9^ did.

### Surgical treatments

After the orientation tests in the local Rybachy field, all birds that showed at least three significant control directions were divided into two experimental groups with the same number of individuals. To avoid any directional biases in our experimental groups, we ranked all the birds’ azimuths on a circular diagram from 0° to 359° clockwise and assigned each bird with an odd rank to one group and each bird with an even rank to another one. One group – *real V1-sectioned birds* – underwent bilateral section and removal of approximately 3–5 mm of the ophthalmic branch of the trigeminal nerve (V1). Surgical removal of a part of V1 has been used in various previous studies^17,18,20,21^, since it represents the only reliable method to prevent any sensory input from V1 from reaching the brain. The absence of re-growth was confirmed in previous studies^17^. Surgical removal of a part of V1 has been shown to not affect the birds’ magnetic compass^17,21^. To make sure that birds were visually undistinguishable as to their treatment and to test for any effect on the birds´ behaviour by the surgery itself, another group – the *sham-sectioned birds* – underwent exactly the same surgical procedure except that V1s were left intact. To perform the surgeries, all birds were anaesthetized using intramuscular injection of Medetomidine (Domitor©, 0.1%; 0.1 ml/kg body weight) +Ketamine (10%; 0.1 ml/kg body weight) and immobilized using a custom built holder. Access to V1 was gained through a small incision along the dorsal rim of the orbit and gentle retraction of the eyeball and oculomotor muscles. To minimize the duration of anaesthesia, the effect of Medetomidine was antagonized using Atipamezol (Antisedan©, 0.5%; 0.1 ml/kg body weight) at the end of the surgery. Each bird was given at least 3 days to recover from the surgery before participating in any orientation tests. We took great care to perform all the orientation tests and analyses of Emlen funnel data double-blind. For instance, only the person who did the surgical treatments (DH), but not the experimenters who performed orientation tests (AA, AP, NC and DK), was aware which bird belonged to which group.

### Statistics

The nonparametric Mardia-Watson-Wheeler test performed with ORIANA (Kovach Computing Services, version 3.21) was used to test for differences in the mean orientation direction between experimental groups. We did not use the more powerful parametric Watson-Williams test because the r*-*values for our group mean vectors in most cases were <0.75. An r value > 0.75 is a crucial assumption for the Watson-Williams test^37^.

## Electronic supplementary material

Dataset 1
