# Supplementary material for: Magnetic map navigation in a migratory songbird requires trigeminal input
Source: Sci Rep. 2018 Aug 10;8:11975. doi: 10.1038/s41598-018-30477-8 (PMC6086908; doi:10.1038/s41598-018-30477-8)
Supplement: Supplementary file 1 — Dataset 1 [file 41598_2018_30477_MOESM1_ESM.pdf]

Magnetic map navigation in a migratory songbird requires trigeminal input

Alexander Pakhomov, Anna Anashina, Dominik Heyers, Dmitry Kobylkov, Henrik Mouritsen and Nikita Chernetsov

SUPPLEMENTAL DATA: MAGNETIC MAP NAVIGATION IN A MIGRATORY SONGBIRD REQUIRES TRIGEMINAL INPUT

Table S1. Orientation of V1-sectioned reed warblers in each test before (NMF) and after (CMF) virtual magnetic displacement. NA – not active, NS – not significant.

Year	№ bird	NMF									CMF									
										**Mean**										**Mean**
**2013**	**XZ18138**	35°	355°							**25°**	NA	295°								**295°**
**2013**	**XZ18222**	NS	45°							**45°**	NA	NS	8°	NA	NA	NA				**8°**
**2013**	**XZ18330**	235°								**235°**	NS	NA	NA	116°	NA	90°	NS	NA	25	**77°**
**2013**	**XZ18365**	15°								**15°**	300°	NS	NA	NS						**300°**
**2013**	**XZ18380**	115°								**115°**	127°	NS	300°	60°						**42°**
**2013**	**XZ18388**	25°								**25°**	NA	45°	NA	305°						**355°**
**2013**	**XZ18407**	75°								**75°**	NA	0°	NA	NS						**0°**
**2013**	**XZ18430**	NS	355°							**355°**	NA	NS	NA	75°	NA	340°				**27°**
**2013**	**XZ18433**	115°								**115°**	NA	285°	NA	NS	210°	NS				**247°**
**2014**	**XR45687**	15°	25°							**57°**	40°	NS	NS	219°	224°	10°				**40°**
**2014**	**XR45838**	NS	75°	NS	80°					**77°**	100°	NA	136°	NA	92°	101°				**100°**
**2014**	**XR45688**	55°°	NS	NS	NA	NA	NA			**55°**	91°	78°	NA	105°	NS	NS	NA	NA		**91°**
**2014**	**XR45793**	NS	NS	105°	294°					**20°**	NS	NS	199°	40°	220°	NA	NS	NS		**199°**
**2014**	**XR45868**	235°	345°							**290°**	328°	310°	280°	290°	285°	320°	160°	260°		**302°**
**2014**	**XR45880**	NS	105°							**105°**	NS	NS	150°	100°	NS	90°	NS	NS		**113°**
**2015**	**XR56846**	NS	255°	335°	285°	275°	125°	317°	309°	**294°**	345°	NS	89°	140°	NS	NA	NS	NS		**71°**
**2015**	**XR56884**	NS	125°	NS	NA	NA	345°	165°	85°	**105°**	320°	350°	100°	NS	290°	36°	155°	NA		**7°**
**2015**	**XR56909**	315°	NS	NS	265°	NA				**290°**	NS	NA	90°	NA	NS	NA	323°	NS		**27°**
**2017**	**XV03216**	120°	312°	75°	NA					**69°**	NS	NA	5°	170°						**88°**
**2017**	**XV03084**	NS	NS	NS	350°	245°	45°			**344°**	15°	NS	NS	30°						**23°**
**2017**	**XV03115**	335°	NA	NS	NS	75°	NA	325°	NS	**359°**	115°	145°	NS	NS						**130°**
**2017**	**XV03160**	9°	NS	265°	NS	NS	355°			**334°**	230°	NS	NS	NS						**230°**

Table S2. Orientation of sham-sectioned reed warblers in each test before (NMF) and after (CMF) virtual magnetic displacement.

		**NMF**								**CMF**									
**Year**	**№ bird**								**Mean**										**Mean**
**2013**	**XZ18293**	55°	NA						**55°**	NA	340°	NA	NA	NA	NA	NA	NS	NA	**340°**
**2013**	**XZ18375**	5°	NA						**5°**	50°	50°	80°	NS						**60°**
**2013**	**XZ18223**	355°							**355°**	NS	NA	NA	NA	NA	NA	50	299°	300°	**336°**
**2013**	**XZ18268**	112°							**112°**	NA	NS	NS	NA	335°	310°				**322°**
**2013**	**XZ18329**	20°							**20°**	160°	10°	170°	155°	115°	90°				**138°**
**2013**	**XZ18412**	315°							**315°**	0°	6°	NA	NS	NA	80°				**29°**
**2014**	**XR10919**	NS	NS	45°	135°	145°	285°	345°	**62°**	70°	329°	340°	6°	79°					**70°**
**2014**	**XR45483**	NS	107°	NS	44°	80°	83°		**78°**	287°	34°	NA	NA	47°					**287°**
**2014**	**XR45717**	85°	NS	NS	65°				**75°**	153°	NA	NS	110°	NS					**153°**
**2014**	**XR45471**	349°	NS	NA	NS	235°	NS	85°	**358°**	NS	280°	270°	NS	NS	NS	NS	NS		**275°**
**2014**	**XR45614**	NS	NS	125°	265°	NS	125°	125°	**141°**	NS	220°	NS	345°	NS	NS	NS	335°		**282°**
**2014**	**XR45813**	265°	10°	285°	75°				**330°**	200°	110°	230°	125°	NS	NS	250°	NS		**165°**
**2014**	**XR45879**	315°	5°						**340°**	320°	NS	NS	270°	120°	NA	300°	NS		**289°**
**2015**	**XR56773**	325°	NA	77°	NS	345°	NA		**4°**	282°	NS	NS	127°	NS	NS	NS	NA		**160°**
**2015**	**XR56895**	75°	100°						**88°**	70°	260°	NS	NS	NS	11°	340°	NS		**355°**
**2015**	**XR56947**	NS	NS	45°	125°	65°			**78°**	245°									**245°**
**2015**	**XR56927**	NS	145°	280°	65°	40°			**64°**	NS	NS	27°	335°	NS	192°	NA	40°		**354°**
**2017**	**XV03080**	165°	NS	170°	NS				**168°**	NS	155°	NS	330°						**243°**
**2017**	**XV03112**	NS	45°	NA	NA	90°	NS		**68°**	NA	NA	270°	NA						**270°**
**2017**	**XV03222**	NS	NS	130°	345°	145°	NS		**115°**	285°	45°	NS	NS						**345°**
**2017**	**XV03083**	335°	45°	245°	NA				**332°**	40°	250°	0°	310°						**7°**
**2017**	**XV03272**	75°	60°	125°					**86°**	100°	240°	200°	NS						**188°**
**2017**	**XV03284**	85°	140°	55°					**92°**	355°	NA	320°	NS						**337°**
**2017**	**XV03236**	80°	NA	NA	10°	NA			**45°**	10°	345°	NS	NS						**357°**
**2017**	**14287989**	NS	255°	330°	NA	70°	NA		**331°**	20°	310°	295°	NA						**324°**
**2017**	**XV03275**	105°	135°	NS					**120°**	315°	NA	NS	NS						**315°**
**2017**	**XV03206**	35°	325°	NS	NS	NS	NS		**0°**	355°	70°	NS	280°						**355°**
